# Electromagnetic Field Associated With Dermoscope Magnets May Affect the Safety of Cardiac Implanted Electronic Devices Patients

**DOI:** 10.3389/fcvm.2021.757032

**Published:** 2021-10-14

**Authors:** Grzegorz Sławiński, Martyna Sławińska, Zbigniew Usarek, Michał Sobjanek, Maciej Kempa, Aleksandra Liżewska-Springer, Ewa Lewicka, Roman J. Nowicki, Grzegorz Raczak

**Affiliations:** ^1^Department of Cardiology and Electrotherapy, Faculty of Medicine, Medical University of Gdańsk, Gdańsk, Poland; ^2^Department of Dermatology, Venereology and Allergology, Faculty of Medicine, Medical University of Gdańsk, Gdańsk, Poland; ^3^Institute of Nanotechnology and Materials Science, Faculty of Applied Physics and Mathematics, Gdańsk University of Technology, Gdańsk, Poland

**Keywords:** dermoscopy, electromagnetic field, cardiac implantable electronic devices, pacemaker, implantable cardioverter-defibrillator

## Abstract

Dermoscopy is currently used as an auxiliary tool in general dermatology. Since some commercially available dermoscopes have built-in magnets, electromagnetic interference (EMI) may occur when examining cardiac implantable electronic devices (CIED) patients. The aim of the study was to create maps of electromagnetic fields defining a safe distance in terms of EMI. The study was performed in laboratory conditions using measuring equipment specially designed for this purpose. The following dermoscopes have been tested: Illuco IDS-1100, Visiomed Luminis, Visiomed Luminis 2, Heine NC2 with and without a contact plate, DermLite DL4, and DermLite Handyscope. Measurements were made for the following set of lift-off distances: 5, 10, 20, 30, 40, 50, and 150 mm. Each 2D scan consisted of 10-line scans shifted from each other by 10 mm. The strength of the magnetic field decreased with the distance from the faceplate. The distribution of the magnetic field differed depending on the position of the magnets. The highest magnetic field was recorded in the center of the Heine NC2 faceplate (up to 8 mT). In most cases, at a distance of 10 mm, the magnetic field strength was measured below 1 mT, with the exception of Heine NC2 and Heine NC2 with a contact plate. All tested dermoscopes generated a magnetic field of <1 mT at the distance of 20 mm. The use of dermoscopes with built-in magnets may affect the functioning of CIEDs, and the impact may vary depending on the type of dermoscope.

## Introduction

Dermoscopy, apart from its traditional application in the diagnostics of skin neoplasia, is currently used as an auxiliary tool in general dermatology, and the dermoscope is compared to the dermatologist's stethoscope (1). In parallel with the increasing use of dermoscopy, due to the more frequent incidence of skin cancer, the number of patients treated with cardiac implantable electronic devices (CIEDs) is also increasing. CIEDs include cardiac pacemakers, implantable cardioverter-defibrillators (ICDs), and cardiac resynchronization therapy (CRT) devices.

Since some commercially available dermoscopes have built-in magnets, possible electromagnetic interference (EMI) may occur when examining a CIED patient, including sensing disturbances (undersensing or oversensing), asynchronous pacing, increased pacing rate, pacing inhibition, and running the mode switch function. Atrial oversensing may trigger false positive mode switch to an asynchronous mode in dual chamber pacemakers or ICDs. This causes loss of synchrony of atrial and ventricular contractions and associated symptoms such as palpitations, dizziness and a deterioration in exercise tolerance. Atrial oversensing may also trigger inadequate ventricular pacing (2). In contrast, ventricular oversensing can inhibit pacing, a potentially life-threatening condition in a pacemaker-dependent patients (3). In patients with implanted ICD, ventricular oversensing may lead to inadequate therapy (anti-tachycardia pacing or high voltage shock). Inappropriate ICD therapies can be potentially proarrhythmic and harmful, and are associated with worse prognosis (4).

EMIs can also trigger a magnet-like response in the ICD, temporarily or permanently suspending therapy in the event of a threatening ventricular tachyarrhythmia. In addition, on rare occasions, the CIED may switch to a backup mode of operation known as “power-on reset” when the device returns to a back-up pacing mode and enables therapy for ventricular tachyarrhythmia in the ICD (5). So far, only one study has assessed the safety of dermoscopy in the context of EMI, but only for a few of handheld dermoscopes available on the market (6). Additionally, no information was provided on the distribution of electromagnetic fields depending on the position of the magnets in individual dermoscopes.

The aim of the study was to evaluate various types of dermoscopes in laboratory conditions and to create maps of electromagnetic fields defining a safe distance in terms of EMI.

## Materials and Methods

The study was performed from October 1, 2020 to December 18, 2020 in laboratory conditions using measuring equipment specially designed for this purpose. As the study did not include humans, the approval from the Ethics Committee was waived. The measurements were performed by an engineer specializing in magnetic measurements (ZU), assisted by a specialist in cardiac electrotherapy (GS) and a dermatologist who uses a dermoscope in her daily practice (MS).

Dermoscopes with magnets available in Poland, which were provided by two manufacturers, were used for the tests. We tested the following dermoscopes: Illuco IDS-1100 (Illuco Corporation, South Korea), Visiomed Luminis (Canfield Scientific, USA), Visiomed Luminis 2 (Canfield Scientific, USA), Heine NC2 with and without a contact plate (HEINE Optotechnik GmbH & Co. KG, Germany), DermLite DL4 (3Gen Inc., USA), and DermLite Handyscope (3Gen Inc., USA). The MPR-H2 (MagLabs s.c, USA) magnetometer was used to measure the magnetic field generated by a dermoscope. The main part of the magnetometer is a probe consisting of several Hall sensors, but for the purpose of this study only one of them was used to measure the magnetic field. Using a magnetometer, the magnetic field was measured, which is perpendicular to the faceplate of the dermoscope. The probe was additionally equipped with a sensor (encoder) to measure the probe displacement through the magnetic field. The distance (*x* direction) was measured with a resolution of 1.12 mm. The magnetometer allowed to measure the magnetic flux density in two ranges: 2 mT with a resolution of 1 μT and 20 mT with a resolution of 10 μT. The first range was used when the output power of the magnetometer did not exceed 2 mT. A measurement platform was created that allowed to maintain a constant distance between the magnetometer and the faceplate. This distance was called the lift-off distance. Two-dimensional (2D) magnetic field scans were performed for each dermoscope at different lift-off distances. The measurements were made for the following lift-off distances: 0, 5, 10, 20, 30, 40, 50, and 150 mm. Each 2D scan consisted of 10-line scans shifted from each other by 10 mm. Therefore, the resolution of the scans performed is different for the *x* direction (1.12 mm) and for the *y* direction (10 mm). The obtained data was collected and saved using a virtual instrument created in the LabVIEW environment. The raw data was then processed using Python 3.0 libraries. The planned endpoint of the study was to demonstrate a safe distance that would allow dermoscope examination of a patient with an implanted CIED.

## Results

**Figure 1:**
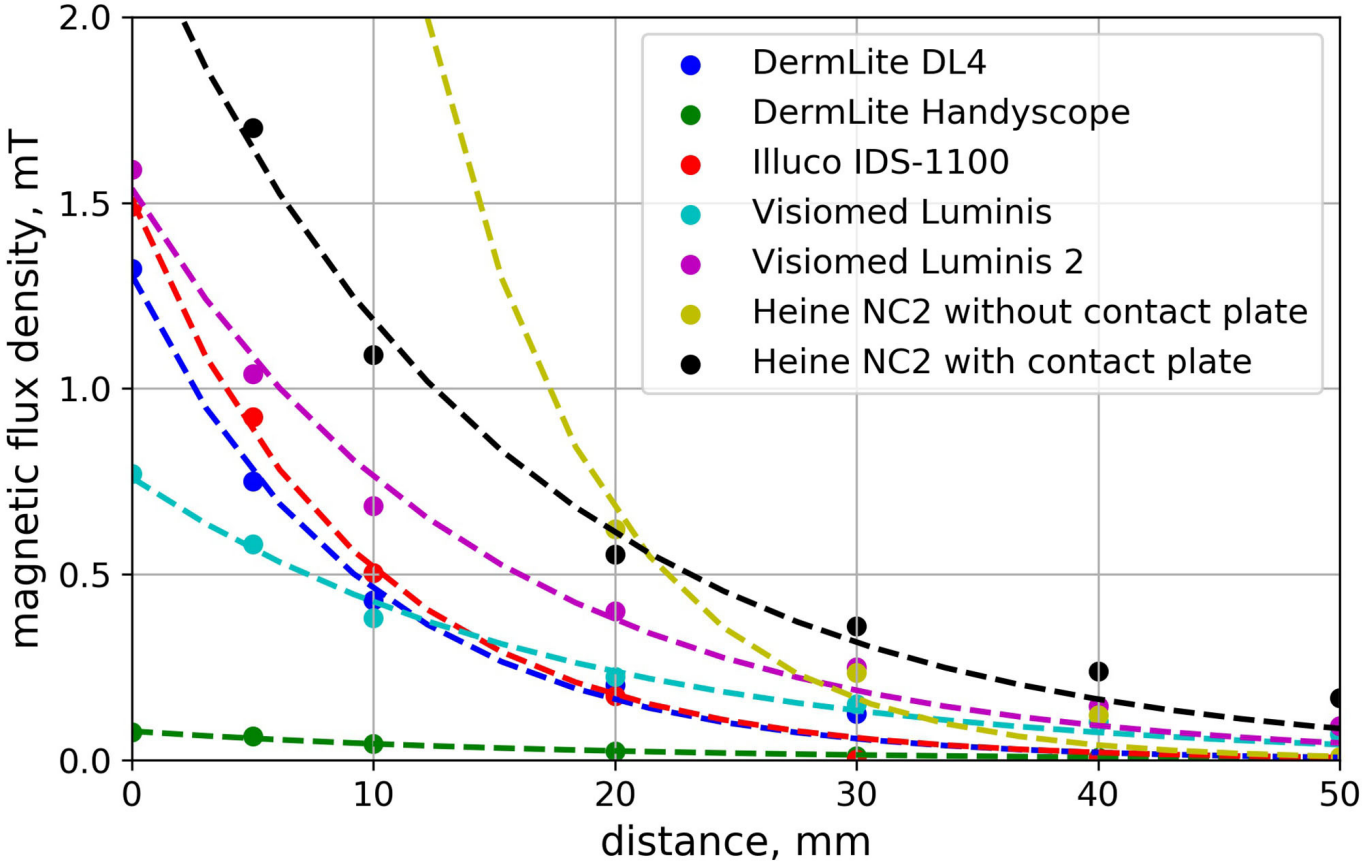
Dependence of the magnetic flux density on the distance from the dermoscope faceplate. Each curve was determined for a path that starts at the point over a faceplate corresponding to the maximum absolute value of measured magnetic flux density.

**Figure 2:**
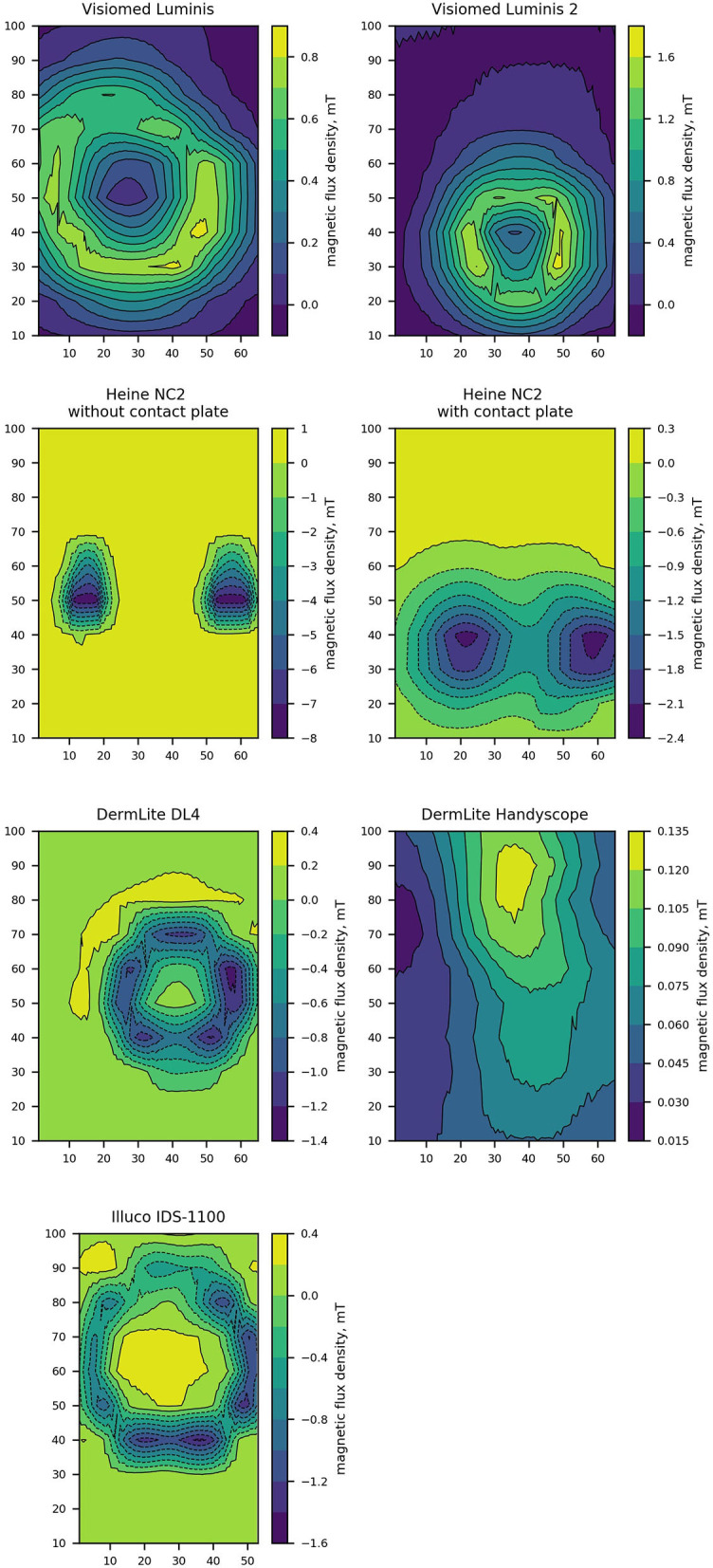
Contour plots representing the perpendicular component of magnetic flux density measured directly over a dermoscope faceplate of the tested dermoscopes models. Dimensions of contour plots are expressed in milimeters.

**Figure 3:**
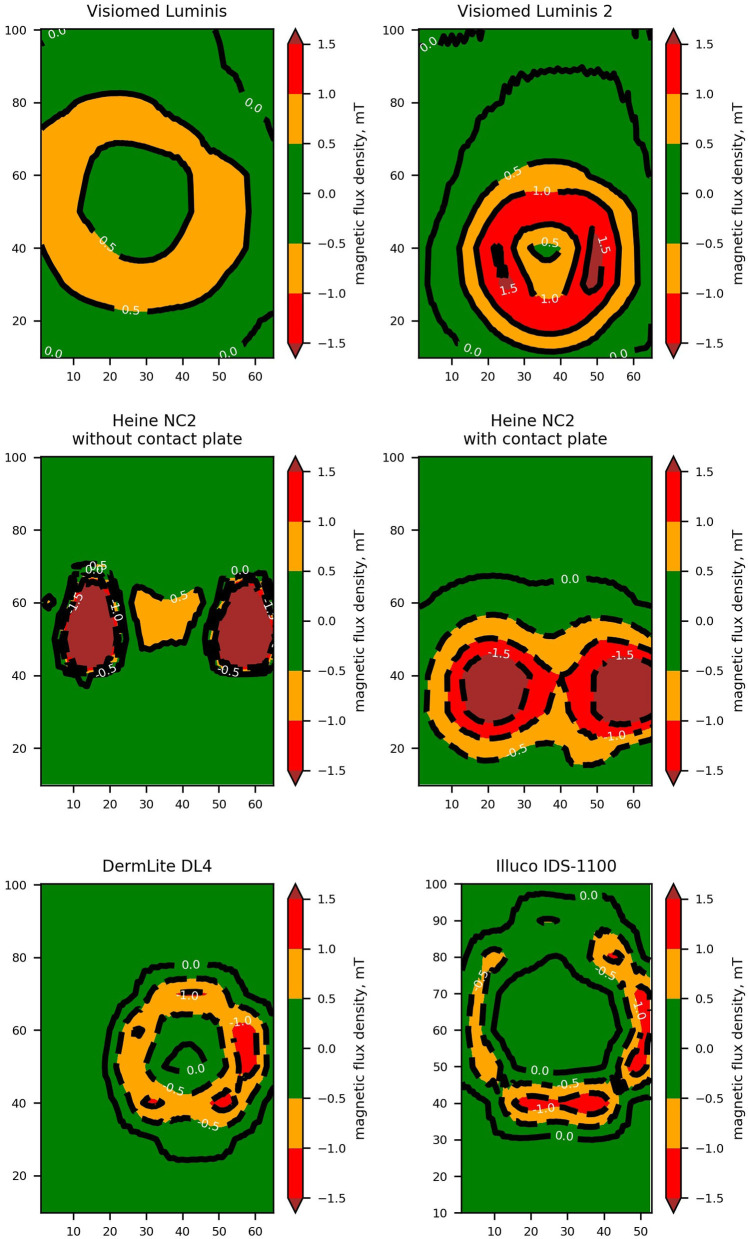
High contrast contour plots representing the perpendicular component of magnetic flux density measured directly over a dermoscope faceplate. Both axes of the contour plots are labeled with a dimension expressed in milimeters. Areas where the magnetic field exceeds thresholds of 0.5, 1.0, and 1.5 mT are highlighted.

The strength of the magnetic field decreased with the distance from the faceplate (Figure 1, Table 1). The magnetic field distribution measured at the faceplate differed depending on the arrangement of magnets in a given device (Figures 2, 3). For most devices magnets are arranged on the circumference of the faceplate. Magnetization of the magnets is generally directed perpendicularly to the faceplate. As a result, the highest value of the magnetic flux density is usually not observed in the center but on the periphery of the faceplate. This is confirmed by the results presented in Figure 2, in particular for Visiomed Luminis, Visiomed Luminis 2, Dermlite DL4, and Illuco IDS-1100. The highest magnetic field was recorded for the Heine NC2 (up to 7.8 mT) directly above its two magnets. The circumferential field distribution is not visible with the Dermlite Handyscope. The reason for this is the long distance (several cm) of the magnets that attach this device to a smartphone, from the faceplate. At this distance, the magnetic flux is diffused and its density significantly decreases. The Visiomed Luminis and DermLite Handyscope were characterized by the lowest density of the generated magnetic flux and as the only two they did not exceed the value of 1 mT (at the distance of 0 mm), which is indicated by CIEDs manufacturers as the threshold value for activating the magnetic switch in CIEDs. In most cases, the magnetic field strength at a distance of 10 mm was below 1 mT, except for the Heine NC2 and Heine NC2 with a contact plate. All tested dermoscopes generated a magnetic field of <1 mT at a distance of 20 mm. The safe lift-off distances for the magnetic flux density of 0.5, 1.0, and 1.5 mT differed between the tested dermoscopes (Table 2). Therefore, on the basis of the obtained results, the distance ensuring the highest probability of safe examination of a patient with an implanted CIED (assuming a lower EMI threshold—i.e., 1.0 mT and regardless of the type of dermoscope) is 17 mm from the implanted CIED.

**Table 1:** The strength of the magnetic field depending on the type of dermoscope and distance from the faceplate.

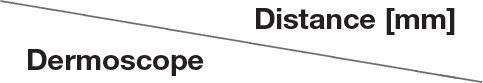	**0**	**5**	**10**	**20**	**30**	**40**	**50**	**150**
Dermlite DL4	1.322	0.750	0.431	0.202	0.124	0.006	0.002	−0.003
Dermlite handyscope	0.075	0.064	0.044	0.023	0.011	0.007	0.005	0.014
Illuco IDS-1100	1.490	0.924	0.505	0.173	0.001	0.004	−0.004	0.003
Visiomed Luminis	0.772	0.580	0.383	0.225	0.150	0.102	0.069	0.011
Visiomed Luminis 2	1.589	1.040	0.684	0.402	0.250	0.145	0.092	0.009
Heine NC2 without contact plate	7.765	7.766	2.741	0.621	0.236	0.121	0.009	0.001
Heine NC2 with contact plate	2.283	1.701	1.091	0.554	0.361	0.239	0.167	0.015

*Values of the magnetic flux density in the table are expressed in mT*.

**Table 2:** The distance from the dermoscope faceplate in a vertical plane at which the magnetic flux density of 0.5, 1.0, and 1.5 was measured.

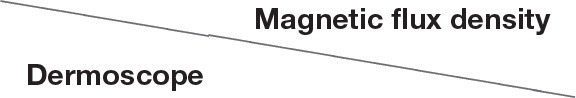	**1.5 mT**	**1.0 mT**	**0.5 mT**
DermLite DL4	N/A	3 mm	9 mm
DermLite Handyscope	N/A	N/A	N/A
Illuco IDS-1100	N/A	4 mm	10 mm
Visiomed Luminis	N/A	N/A	7 mm
Visiomed Luminis 2	N/A	6 mm	16 mm
Heine NC2	14 mm	17 mm	22 mm
Heine NC2 with contact plate	6 mm	13 mm	23 mm

*The values of magnetic flux density provided by manufacturers which may affect the functioning of the CIED are 1.0 mT (in Medtronic, Boston Scientific, Abbott devices) and 1.5 mT (Biotronik).*

*N/A, not applicable*.

## Discussion

As the indications for treatment with CIEDs expand, the number of these devices continues to increase. In Western Europe, in 2005–2011, the number of implanted pacemakers increased from 82.9/100,000 to 93.8/100,000, and CRT from 6.0/100,000 to 14.0/100,000 (7). The number of patients treated with ICD has also increased significantly. Between 1993 and 2009, the number of ICD implantations in the United States increased from 6.1/100,000 to 46.2/100,000 (8). At the same time, the incidence of skin cancer and skin melanoma is increasing, therefore, it is very likely that patients with implanted CIED may require examination with a dermoscope (9). There are also reports in the literature of malignant skin neoplasms developing around the implanted CIED, including melanoma, basal cell carcinoma, leiomyosarcoma, and atypical fibroxanthoma (10–12).

Some dermoscopes have built-in magnets, e.g., to facilitate the process of connecting and disconnecting the contact plate or compatible electronic devices which are required for their use, such as smartphones or tablets. The electromagnetic field generated by these magnets may interfere with the operation of the CIED, which may pose a risk to pacemaker-dependent patients (pacing inhibition) and to ICD patients (inhibition of ventricular tachyarrhythmia detection). CIEDs contain a magnetic switch (reed switch) which is activated by a sufficiently strong magnetic field. The reed switch consists of two metal magnetic strips in a glass capsule. It was designed to prevent the adverse effects of the magnetic field. The most common configuration is to separate these strips (open-switch) from successive contacts of the strips (closed-switch) after exposure to a strong magnetic field. This changes the voltage detected by the amplifier in the CIED, which causes the device to switch to the specified program (13). According to information provided by CIED manufacturers, the reed switch (starts to close at a field strength of 1.0 mT (in Medtronic, Boston Scientific, Abbott devices) or 1.5 mT (Biotronik), which means that stronger static magnetic fields may affect the functioning of the CIED (14).

With regard to dermoscopes with integrated magnets, so far only Rishpon et al. (6) assessed in laboratory conditions their potential impact on the performance of CIEDs. The authors tested the following devices: DermLite DL4 and DL4w [3Gen], VEOS HD1 and HD2 [Canfield Scientific Inc], and NC1 and NC2 [Heine Optotechnik]. In their study, the authors used the gauss (G), which is a unit of measurement of magnetic flux density (1 G = 0.1 mT). According to the study the magnetic field strength at the faceplate varied between 2.22 and 9.98 G (respectively, 0.222 and 0.998 mT). At the distance of 0.5 cm it varied between 0.82 and 2.4 G (respectively, 0.082 and 0.024 mT); at 1.0 cm between 0.5 and 1.04 G (respectively, 0.05 and 0.104 mT), and 0 G for all devices at 15 cm. The values obtained by the authors in this study for DermLite DL4 dermoscope and Heine NC2 were significantly lower in comparison to the results obtained in our study. The differences may be explained by a different methodology, i.e., the lack of contour maps of the electromagnetic field above the surface of the dermoscope and relying solely on the measurements in its central part—which, as we confirmed in our study, does not always correspond to a place with maximum magnetic flux density. In our study, the maximum magnetic flux density was 7.8 mT for the Heine NC2 dermoscope without a contact plate. However, in most of the measurements it exceeded the safety threshold values provided by CIED manufacturers. Importantly, based on our research, no dermoscope exceeded the magnetic flux density of 10 mT, i.e., the threshold activating the AutoDetect function in CIED designed to detect a strong magnetic field and allowing for the safe performance of an MRI examination. Activating this function would be particularly dangerous as it would prevent detection of ventricular arrhythmias (15).

Among the three currently used dermoscopy techniques (standard contact dermoscopy, polarized contact dermoscopy, and polarized non-contact dermoscopy), only contact dermoscopy requires direct contact with the patient's skin. In contrast, non-contact dermoscopes with built-in cross-polarized filters do not require direct skin contact and have been suggested to be safer for patients with CIEDs (6). Similarly to Rishpon et al. (6), we found that a distance of only a few millimeters from a CIED can significantly reduce the strength of the magnetic field. In addition, we showed significant differences in the magnetic field in the spatial presentation for individual models of dermoscopes, which results from the different arrangement of magnets in these devices. It is necessary to remember about these differences when examining skin lesions not only directly over the implanted CIEDs, but also in the close vicinity of the CIEDs. In our study, among the investigated dermoscopes with embedded magnets, the DermLite Handyscope had the best safety profile, but it should be emphasized that using it requires a connection to a smartphone or tablet, which in certain conditions may electromagnetically interfere with the CIEDs. This may be the subject of a future study in which different modern devices connected to the dermoscope should be used, taking into account different scenarios, e.g., active Wi-Fi connection, data transfer via Bluetooth, etc.

Many medical devices and procedures can interfere with the functioning of CIEDs. These include magnetic resonance imaging, left ventricular assist devices, radiotherapy, electrical cardioversion, radiofrequency ablation, electrocoagulation, percutaneous nerve electrostimulation, lithotripsy, and electroconvulsive therapy (16). The potential risk of electromagnetic disturbances during dermatological procedures was also included in the guidelines published by the British Society of Dermatological Surger*y* (17). It is recommended that procedures performed within 5 cm from the CIED take place in the presence of a cardiologist and with a programmer for a given CIED in case of any problems. There are also single reports confirming safety of smartwatches and their chargers, smartphones (18), electric cars (19), induction ovens, and body scanners among patients with implanted CIEDs. More disturbing data came from a study by Lee et al. (20) assessing the safety of portable headphones in patients with CIEDs. In this study headphones generated a magnetic field stronger than 20 mT. This resulted in disturbances in the functioning of CIED in as many as 30% of patients.

Despite the fact that dermoscopes may influence CIED functioning, it is important to balance the benefits and risks of performing dermoscopic examination in this group of patients. In fact, dermoscopy is a method used mainly for diagnostics of potentially life-threatening skin tumors, while to date we have not found any case reports describing documented CIED functioning disturbances that occurred during dermoscopic examination. From the other side, such potential risk cannot be neglected, which has been clearly shown in our study. Increasing the awareness between both clinicians and patients seems to be crucial. Medical professionals using dermoscopes in daily practice should be aware of the presence of magnets in devices they use and their possible interactions with CIED. Before using a dermoscope with a magnet, the question about previous CIED implantation should be asked routinely. Patients undergoing CIED implantation should be educated by cardiologists about the potential risk associated with dermoscopes magnets and report the fact of having an implanted CIED to the examining dermatologist. It is important to underline the fact that having an implanted CIED does not disqualify the patient from a dermoscopic examination. Use of another model without embedded magnets is completely safe and does not affect CIED in any way. If the physician possesses only the device with embedded magnets, it would be advisable not to cross the safety distance which depends not only on the dermoscopic device but also on CIED type. In case of need to examine the skin just over the CIED, patient referral to another specialist for examination with a safe dermoscopic device would be recommended.

## Limitations of the Study

The main limitation of the study is its laboratory setting, without human subjects with CIEDs and not validated or tested in cardiac devices. However, due to the scarce data in the literature, the assessment of the hypothetical risk of EMI among patients with implanted CIEDs is an important preliminary study confirming the need for further studies in the field. Another limitation is the fact that we did not have an opportunity to confirm the measurements on multiple devices of the same model of dermoscope, what would be one of the issues to be addressed in future studies.

## Conclusions

In conclusion, it should be emphasized that the use of dermoscopes with built-in magnets may affect the functioning of the CIED, with significant differences between individual devices. Physicians who use dermoscopes in their daily practice should be aware of the presence of magnets in these devices and be aware of the possible consequences this may have in patients with pacemakers, ICDs and CRT devices. Another aspect is the use of dermoscopes with built-in magnets in conjunction with smartphones and tablets, and their possible impact on the functioning of CIED, which requires further studies.

## Data Availability Statement

The raw data supporting the conclusions of this article will be made available by the authors, without undue reservation.

## Author Contributions

GS, MSł, ZU, MSo, MK, AL-S, and EL conceived and planned the experiments. GS, MSł, and ZU carried out the experiments. GS, MSł, ZU, MSo, MK, AL-S, EL, RN, and GR contributed to the interpretation of the results. GS, MSł, and ZU took the lead in writing the manuscript. All authors provided critical feedback and helped to shape the research, analysis, and manuscript.

## Conflict of Interest

The authors declare that the research was conducted in the absence of any commercial or financial relationships that could be construed as a potential conflict of interest.

## Publisher's Note

All claims expressed in this article are solely those of the authors and do not necessarily represent those of their affiliated organizations, or those of the publisher, the editors and the reviewers. Any product that may be evaluated in this article, or claim that may be made by its manufacturer, is not guaranteed or endorsed by the publisher.
